# Draft Genome Sequence of *Aeromonas* sp. Strain EERV15

**DOI:** 10.1128/genomeA.00811-16

**Published:** 2016-08-18

**Authors:** Elham Ehsani, Israel Barrantes, Johanna Vandermaesen, Robert Geffers, Michael Jarek, Nico Boon, Dirk Springael, Dietmar H. Pieper, Ramiro Vilchez-Vargas

**Affiliations:** a Center for Microbial Ecology and Technology (CMET), University of Ghent, Ghent, Belgium; b Microbial Interactions and Processes Research Group, Helmholtz Centre for Infection Research, Braunschweig, Germany; c Department of Earth and Environmental Sciences, KU Leuven, Belgium; d Genome Analytics, Helmholtz Centre for Infection Research, Braunschweig, Germany

## Abstract

We report here the draft genome sequence of *Aeromonas* sp. strain EERV15 isolated from sand filter. The organism most closely related to *Aeromonas* sp. EERV15 is *Aeromonas veronii* B565, with an average 83% amino acid sequence similarity of putatively encoded protein open reading frames.

## GENOME ANNOUNCEMENT

*Aeromonas* spp. are present in a wide range of habitats (1). These bacteria are associated with an aquatic environment and can be isolated from different environmental sources, such as food, invertebrates, fish, birds, ticks, insects, domesticated pets, and natural soils (2–4). *Aeromonas* spp. are classified as mesophilic or psychrophilic. The mesophilic group, comprising *Aeromonas hydrophila*, is associated with human infections, whereas the psychrophilic group, comprising *Aeromonas salmonicida* as the only species was associated so far with diseases in fish (5). *Aeromonas* clinical infections are divided into four categories (5), which are gastrointestinal tract syndromes (6, 7), wound and soft tissue infections (8, 9), blood-borne dyscrasias (10, 11), and miscellaneous infections (5, 12, 13). Most clinical infections are caused by *A. hydrophila*, *A. caviae*, and *A. veronii* biovar sobria (14). Here, we announce the draft genome sequence of *Aeromonas* sp. strain EERV15 (taxon ID: 1833892), previously named *Aeromonas* sp. strain K62 (J. Vandermaesen, B. Lievens, D. Springael, submitted for publication).

The genome was sequenced using the Illumina MiSeq platform, which generated paired-end read sequences of 250 bp. These were assembled using Edena (15, 16), producing 207 contigs, with a total genome size of 4,464,577 bp (58% G+C content; *N*_50_, 173 kbp) and a coverage average of 200×. Automatic annotation was performed using the RAST server version 4.0 (17), generating 4,017 potentially protein-coding genes (open reading frames [ORFs]).

In order to find the closest related strain to EERV15 at the genome level, a comparison between the draft genome of *Aeromonas* sp. strain EERV15 and 33 genomes or draft genomes (six *Aeromonas* isolates, HZM [18], ZOR0001 [accession no. PRJNA205571], ZOR0002 [accession no. PRJNA205572], L1B53 [accession no. PRJNA270791], 159 [19], and MDS8 [20]; 10 *Aeromonas veronii* strains, AMC34 [accession no. PRJNA71515], AMC35 [accession no. PRJNA71519], Hm21 [21], AER39 [accession no. PRJNA71513], AER397 [accession no. PRJNA71517], B565 [22], CECT4257 [accession no. PRJEB7044], CECT4486 [accession no. PRJEB7050], CIP 107763 [accession no. PRJEB7047], and *A. veronii* biovar sobria [accession no. PRJEB7051]; 10 *Aeromonas hydrophila* strains, J-1 [accession no. PRJNA227242], YL17 [accession no. PRJNA234473], NJ-35 [accession no. PRJNA226230], pc 104A [23], Ae34 [24], ML09-119 [25], 4AK4 [26], ATC7966 [27], AL09-71 [28], and AL06-06 [29]; two *Aeromonas salmonicida* strains, CBA100 [accession no. PRJNA257751] and *A. salmonicida* subsp. *pectinolytica* 34mel [30]; together with *A. rivuli* DSM 22539 [accession no. PRJEB7035], *A. enteropelogenes* CECT4487 [accession no. PRJEB7028], *A. dhakensis* CECT7289 [accession no. PRJEB7020], *A. diversa* CECT4254 [accession no. PRJEB7026], and *A. media* WS [31]) was performed. The results showed that the closest related strain to EERV15 is *Aeromonas veronii* strain B565, with an 83% average similarity of ORFs. The two strains share 3,371 ORFs with >80% similarity, and 390 ORFs observed in the genome of the strain EERV15 were absent from the genome of strain B565.

Strain EERV15 harbors two copies of the high-persistence gene *hipA* and one copy of the *hipB* gene (32, 33), similar to those found in *A. media* WS, whereas in four *A. hydrophila* strains (pc 104A, ML09-119, NJ-35, and AL09-71), only *hipA* was detected. The *hipA* gene was found to be an important persistence factor in *Escherichia coli* inducing dormancy, while the *hipB* gene neutralizes the effect of the *hipA* gene. The *hipA* gene cannot be expressed in the absence of *hipB* because of its deleterious effects on cell growth (34, 35). Thus, strain EERV15 potentially possesses the ability to be persistent against antibiotics inducing dormancy.

### Accession number(s).

This draft genome sequencing project has been deposited at EMBL-EBI European Nucleotide Archive (ENA) under the accession numbers FKKY02000001 to FKKY02000207.
